# Basis function model to extract the combined confocal and fall-off function from multiple optical coherence tomography A-scans

**DOI:** 10.1117/1.JBO.30.2.025003

**Published:** 2025-02-26

**Authors:** Daniel J. Phan, Martin Were, Jörn-Hendrik Weitkamp, Audrey K. Bowden

**Affiliations:** a Vanderbilt University, Vanderbilt Biophotonics Center, Department of Biomedical Engineering, Nashville, Tennessee, United States; b Vanderbilt University Medical Center, Vanderbilt Institute for Global Health, Department of Biomedical Informatics and Medicine, Nashville, Tennessee, United States; c Vanderbilt University Medical Center, Department of Pediatrics, Nashville, Tennessee, United States; d Vanderbilt University, Department of Electrical and Computer Engineering, Nashville, Tennessee, United States

**Keywords:** optical coherence tomography, confocal, fall-off, basis functions

## Abstract

**Significance:**

Many derivatives of optical coherence tomography (OCT) rely on the depth-dependent information of the sample in the image. System depth-dependent effects, such as the confocal effect and the sensitivity fall-off, should be corrected to improve the accuracy of the images and information derived from them.

**Aim:**

We developed a new single-shot method to extract the combined confocal and fall-off functions and remove system-generated depth-dependent effects from OCT images.

**Approach:**

The combined function is modeled as a linear combination of basis functions whose coefficients are found from two or more A-scans (or B-scans) of a sample that are vertically shifted within the imaging range. No prior knowledge of the OCT system parameters or assumed form for the confocal and fall-off functions is needed.

**Results:**

The method was derived and validated with simulations and OCT images of a phantom, a biological sample, and human retina. Improvement over the Ratio Fit method was demonstrated.

**Conclusions:**

The improvement in the extraction of the combined confocal and fall-off effects by this method should lead to improved medical diagnosis through more accurate attenuation coefficient calculations. The method enables future applications of OCT where precise removal of all depth-dependent effects on OCT images is critical.

## 1 Introduction

Extracting the depth-dependent attenuation coefficient (AC) from optical coherence tomography (OCT) data is useful in aiding medical diagnosis of various clinical diseases such as age-related macular degeneration, glaucoma, and bladder cancer.^1^^,^^2^ To enable accurate extraction of the AC, depth-dependent system effects from OCT A-scans, such as the confocal effect and the sensitivity roll-off or fall-off, must be corrected.^2^^–^^4^ The confocal effect is related to the focusing of Gaussian beams, affecting the transverse resolution and depth of focus. Sensitivity fall-off, sometimes referred to as sensitivity roll-off or just fall-off, is the observed decrease in OCT sensitivity at greater imaging depths. Both effects manifest as “envelopes” on a typical A-scan in spectral domain OCT (SD-OCT) systems. When performing image corrections, the confocal function and fall-off function are normally estimated separately, but these estimations may require information about the system, such as the spectral resolution, wavelength-per-pixel of the spectrometer, and the central spectral frequency of the laser source, which is not always readily available.^4^ In addition, the prescribed functions used to model the two effects contain limited degrees of freedom, suggesting the potential for an improved model to fit the data better. An improved model may be helpful, for example, in the case where the confocal function is sample-dependent due to variations in the refractive indices within a sample.^3^

The confocal function can be measured experimentally by placing a mirror in the sample arm and recording the A-scan at multiple depths. However, this method requires multiple A-scans to approximate the confocal function.^5^ Moreover, it cannot account for sample-dependent effects on the confocal function.

Alternatively, a common computational method to extract the confocal function from a system with unknown parameters is to use information from two B-scans of the sample taken at different positions relative to the focus of the light.^6^^,^^7^ This method, referred to here as “Ratio Fit,” assumes that the fall-off function is either negligible or can be determined and removed before confocal function extraction. In this method, the model of the confocal function takes two parameters: the depth of the focal plane and the apparent Rayleigh range.^3^^,^^6^^–^^8^ Notably, this assumed model may not be adequate in practice. For example, it can depend on the refractive index and the effects of scattering on the Rayleigh range within the sample, which may require that additional variables be added to the model.^9^ “Spectral Ratio Fit” is similar to Ratio Fit, but it uses sub-spectral A-scans (i.e., A-scans derived from the short-time Fourier transform) from the interferogram of a single A-scan to determine the focus position that defines the confocal function, instead of using multiple A-scans with different focus or sample positions.^8^ An advantage of this method is that it only requires a single A-scan and can account for wavelength-dependent Rayleigh lengths. However, this method still suffers from the same shortcoming as standard Ratio Fit in that it incorrectly assumes a specific, potentially inaccurate, form of the confocal function. This method also requires the correction of fall-off as a separate step before extracting the confocal function.

An alternative approach to the fit methods finds the confocal function parameters and total AC at the same time by assuming different forms of the OCT intensity profile as a function of depth.^10^ Although this approach allows for different signal models, including single and multiple scattering, the accuracy of the results still depends on the validity of the assumed model. As in most publications, the standard form of the two-parameter confocal function, as assumed in Ratio Fit, is incorporated into the OCT signal models.

Similar to extraction of the confocal function, extraction of the fall-off function can also be experimentally time-consuming and computationally inaccurate. The effects of fall-off can be modeled by several prescribed functions of increasing complexity. The simplest Gaussian model requires measurement of the system sensitivity using a mirror sample placed at several imaging depths.^11^ This approach requires additional data collection outside of the primary sample data collection, which is not always possible or practical, especially in a busy clinical workflow. Other, more complex models require prior knowledge about the OCT system such as the central wavelength of the light source, the spectral resolution, and the sampling resolution.^12^^,^^13^ Again, these system parameters are not necessarily known or easily obtainable, particularly in clinical contexts.

In this study, we introduce “Basis Fit” as a new strategy to provide depth-dependent correction for both confocal and fall-off effects simultaneously. Notably, we consider for the first time that it is not necessary to separately correct for both effects; rather, the correction can be combined into a single operation and represented as a single function. Here, we model the function as a linear combination of basis functions whose coefficients are found by the fit of the model to the experimental data derived from two A-scans of the same sample that are vertically shifted from each other, as would be obtained in the standard Ratio Fit method. The ability to extract both the confocal and fall-off simultaneously reduces the overhead of having to determine each separately, eliminates the need to find the coefficients that define each of the two effects, and automatically accounts for other depth-dependent effects that the user is unaware of (e.g., sample-dependent dispersion or other focal chromatic effects). Hence, Basis Fit is particularly useful when prior knowledge of the system parameters is not available or cannot be easily obtained (e.g., as is appropriate for commercial and clinical systems). Moreover, the flexibility of the model to fit the data allows for more accurate correction across the image in cases where the confocal function may not be constant for all A-scans in a B-scan, such as when performing imaging with high numerical-aperture lenses.^3^ We show through simulation and analysis of clinical data that Basis Fit leads to more accurate calculation of AC values in real systems, where the assumptions of Ratio Fit are limiting.

## 2 Background

Basis Fit models confocal and fall-off effects as a single function that is a linear combination of basis functions. The coefficients of this linear combination are determined from the ratio of the intensities of two A-scans of the sample that are vertically shifted in depth. Consider the case where the system focus is fixed and only the sample is vertically shifted. Let h(z) represent the system confocal function, f(z) its fall-off function, and g(z) a combined function

g(z)=h(z)f(z), (1)

where z is depth, typically in mm. Let $A_{1}(z)$ and $A_{2}(z)$ represent the ideal intensity profiles of the OCT signal that are free from confocal and fall-off effects. The measured (i.e., uncorrected) A-scans $I_{1}(z)$ and $I_{2}(z)$ affected by the confocal and fall-off functions are therefore

$$I_{1}(z)=g(z)A_{1}(z),$$ (2)

and

$$I_{2}(z)=g(z)A_{2}(z).$$ (3)

## 3 Theory

### 3.1 Mathematical Derivation

In this derivation, we assume that $I_{1}(z)$ and I(z) computed from the raw OCT interferogram have already gone through the standard post-processing steps, including, but not limited to background subtraction, k-linearization, and dispersion correction. If the sample is vertically shifted by Δz such that $A_{2}(z)$ is deeper than $A_{1}(z)$, then

$$A_{2}(z)=A_{1}(z-\Delta z),$$ (4)

or equivalently,

$$A_{2}(z+\Delta z)=A_{1}(z).$$ (5)

Therefore, from Eqs. (3) and (5),

$$I_{2}(z+\Delta z)=g(z+\Delta z)A_{2}(z+\Delta z)=g(z+\Delta z)A_{1}(z).$$ (6)

The ratio of the corresponding intensities of the two uncorrected A-scans is Eq. (2) divided by Eq. (6). Substituting $A_{1}(z)$ for $A_{2}(z+\Delta z)$ into the ratio expression produces

$$\frac{I_{1}(z)}{I_{2}(z+\Delta z)}=\frac{g(z)A_{1}(z)}{g(z+\Delta z)A_{2}(z+\Delta z)}=\frac{g(z)A_{1}(z)}{g(z+\Delta z)A_{1}(z)}.$$ (7)

Cancelling out $A_{1}(z)$ from Eq. (7) produces a relationship between the ratios of the intensities of the two A-scans and the ratio of the combined function g(z) and a vertically shifted version of itself

$$\frac{I_{1}(z)}{I_{2}(z+\Delta z)}=\frac{g(z)}{g(z+\Delta z)}.$$ (8)

Equation (8) equation is the key equation to find g(z). Although inherently nonlinear, in log scale, it becomes linear. In dB-scale, Eq. (8) is

$$10\;\log_{10}\;I_{1}(z)-10\;\log_{10}\;I_{2}(z+\Delta z)=10\;\log_{10}\;g(z)-10\;\log_{10}\;g(z+\Delta z).$$ (9)

Note that because $I_{1}(z)$ and $I_{2}(z)$ are intensity profiles, the squares of the amplitudes after the Fourier transforms of their interferograms, $10\;\log_{10}$, instead of $20\;\log_{10}$, is used to convert them into dB-scale. Let $10\;\log_{l0}\;g(z)$ be approximated by the basis functions $\beta_{1}(z),\beta_{2}(z),\ldots ,\beta_{N+1}(z)$. Then,

$$10\;\log_{10}\;g(z)\approx c_{1}\beta_{1}(z)+c_{2}\beta_{2}(z)+\ldots +c_{N+1}\beta_{N+1}(z),$$ (10)

where (N+1) is the number of basis functions needed to model $10\;\log_{l0}\;g(z)$. The coefficients $c_{1},c_{2},\ldots ,c_{N+1}$ are to be determined. We have (N+1) basis functions here because as shown later, the coefficient $c_{1}$ cannot and need not be determined, and only N coefficients remain. Let y(z) denote the left-hand-side of Eq. (9)

$$y(z)=10\;\log_{10}\;I_{1}(z)-10\;\log_{10}\;I_{2}(z+\Delta z).$$ (11)

The difference in intensity, y(z), can be computed from two vertically shifted intensity A-scans and is referred to as “data” in this paper. Then,

$$\begin{array}{c} y(z)=[c_{1}\beta_{1}(z)+c_{2}\beta_{2}(z)+\ldots +c_{N+1}\beta_{N+1}(z)]\\ -[c_{1}\beta (z+\Delta z)+c_{2}\beta_{2}(z+\Delta z)+\ldots +c_{N+1}\beta_{N+1}(z+\Delta z)]+\;\varepsilon (z)\\ =c_{1}(\beta_{1}(z)-\beta_{1}(z+\Delta z))+c_{2}(\beta_{2}(z)-\beta_{2}(z+\Delta z))\\ +\ldots +c_{N+1}(\beta_{N+1}(z)-\beta_{N+1}(z+\Delta z))+\varepsilon (z), \end{array}$$ (12)

where ε(z) is referred to as the fitting error. In theory, any set of basis functions that can model the shape of the combined function $10\;\log_{l0}\;g(z)$ can be used, such as sine and cosine functions, and monomials. In our case, $10\;\log_{l0}\;g(z)$ is expected to be a simple function in shape because both the confocal function and fall-off function are expected to be simple in shape; thus, the choice of the basis functions is not critical. In this paper, for simplicity, we use Chebyshev polynomials of the first kind, which are related to the sine and cosine functions for a finite interval. Standard Chebyshev polynomials are defined over −1≤x≤1 and denoted as $\beta_{k}(x)$, where k=1,2,…,(N+1)

$$\beta_{1}(x)=1,$$ (13)

$$\beta_{2}(x)=z,$$ (14)

and

$$\beta_{k}(x)=2z\beta_{k-1}(x)-\beta_{k-2}(x).$$ (15)

To proceed with the derivation, the range of the independent variable x in the Chebyshev polynomials $\beta_{k}(x)$ needs to be converted from the interval [−1,1] to z in the interval $\left[0,z_{\max}\right]$, where $z_{\max}$ is the maximing imaging depth of the A-scan. Let these Chebyshev polynomials be denoted as $\beta_{k}(z)$. The first basis function $\beta_{1}(z)=1$, so that $\beta_{1}(z)-\beta_{1}(z+\Delta z)=0$. Equation (12) becomes

$$y(z)=c_{2}(\beta_{2}(z)-\beta_{2}(z+\Delta z))+\ldots +c_{N+1}(\beta_{N+1}(z)-\beta_{N+1}(z+\Delta z))+\varepsilon (z).$$ (16)

Note that now there are N coefficients to solve for, from $c_{2}$ to $c_{N+1}$. The right-hand-side of Eq. (16) without the term ε(z) is referred to as “model,” and Eq. (16) is a relationship between the “data” from Eq. (11) and the “model,” with ε(z) serving as the fitting error. The constant $c_{1}$ that corresponds to a proportional constant for g(z) is not present in Eq. (16) and cannot be determined from it. Thus, the combined function g(z) can only be determined up to a scaling factor; therefore, the corrected A-scans can only be determined up to a scaling factor. The scaling factor, however, is just for normalization. Normalization in this paper simply means rescaling the function so that its maximum is one. Different scaling factors shift the intensity curves (in dB) of the corrected A-scans up or down but do not affect the rendering of grayscale OCT B-scan images if the images are rescaled prior to visualization. Equation (16) can be rewritten as

$$y(z)=[c_{2},c_{3},\ldots ,c_{N}][\begin{array}{c} \beta_{2}(z)-\beta_{2}(z+\Delta z)\\ \beta_{3}(z)-\beta_{3}(z+\Delta z)\\ \ldots \\ \beta_{N+1}(z)-\beta_{N+1}(z+\Delta z) \end{array}]+\varepsilon (z).$$ (17)

Note that the above equation is valid at every depth position z in a single A-scan. Writing Eq. (17) for every depth position from $z_{1}$ to $z_{n}$ in a single A-scan, where $z_{1}=0$ and $z_{n}=z_{\max}-\Delta z$, and packaging them into a single matrix equation produces

$$\begin{array}{c} \left[y(z_{1}),y(z_{2}),\ldots ,y(z_{n})\right]\\ =[c_{2},c_{3},\ldots ,c_{N+1}][\begin{array}{c} \beta_{2}(z_{1})-\beta_{2}(z_{1}+\Delta z)\\ \beta_{3}(z_{1})-\beta_{3}(z_{1}+\Delta z)\\ \ldots \\ \beta_{N+1}(z_{1})-\beta_{N+1}(z_{1}+\Delta z) \end{array}|\begin{array}{c} \begin{array}{c} \;\\ \; \end{array}\\ \ldots \\ \begin{array}{c} \;\\ \; \end{array} \end{array}|\begin{array}{c} \beta_{2}(z_{n})-\beta_{2}(z_{n}+\Delta z)\\ \beta_{3}(z_{n})-\beta_{3}(z_{n}+\Delta z)\\ \ldots \\ \beta_{N+1}(z_{n})-\beta_{N+1}(z_{n}+\Delta z) \end{array}]\\ +[\varepsilon (z_{1}),\varepsilon (z_{2}),\ldots ,\varepsilon (z_{n})]. \end{array}$$ (18)

By making appropriate definitions, Eq. (18) can be written as

y=cB+ε, (19)

where

$$y=[y(z_{1}),y(z_{2}),\ldots ,y(z_{n})],$$ (20)

$$c=[c_{2},c_{3},\ldots ,c_{N+1}],$$ (21)

$$B=[\begin{array}{c} \beta_{2}(z_{1})-\beta_{2}(z_{1}+\Delta z)\\ \beta_{3}(z_{1})-\beta_{3}(z_{1}+\Delta z)\\ \ldots \\ \beta_{N+1}(z_{1})-\beta_{N+1}(z_{1}+\Delta z) \end{array}|\ldots |\begin{array}{c} \beta_{2}(z_{n})-\beta_{2}(z_{n}+\Delta z)\\ \beta_{3}(z_{n})-\beta_{3}(z_{n}+\Delta z)\\ \ldots \\ \beta_{N+1}(z_{n})-\beta_{N+1}(z_{n}+\Delta z) \end{array}],$$ (22)

and

$$\varepsilon =[\varepsilon (z_{1}),\varepsilon (z_{2}),\ldots ,\varepsilon (z_{n})].$$ (23)

The row vector c contains the coefficients of the basis functions. The least-squares solution that minimizes the L2 norm of ε is

$$c=yB^{+},$$ (24)

where $B^{+}$ denotes the pseudo-inverse of B.

The solution in Eq. (24) minimizes the fitting error. To visualize how well the model fits the data, we can plot two curves: the data y(z) are given by Eq. (11) and the model is given by the right-hand-side of Eq. (16) without the ε(z) term. This plot is referred to as model-to-data fit. Better alignment of these two curves means better model-to-data fit, which occurs when the two corrected A-scans are closely shifted versions of each other due to improved extraction of g(z).

### 3.2 Implementation

As the implementation of the algorithm is done in pixel space, depth $z_{i}$ is replaced by pixel number i where i=1,2,3,…,n, and vertical shift Δz is replaced by m, where m is now an integer in terms of pixels. Let p denote the pixel index that corresponds to $z_{\max}$, then n=p−m. In pixel units, y and B become

y=[y(1),y(2),…,y(n)], (25)

where $y(i)=10\;\log_{10}\;I_{1}(i)-10\;\log_{10}\;I_{2}(i+m,)$, and

$$B=[\begin{array}{c} \beta_{2}(1)-\beta_{2}(1+m)\\ \beta_{3}(1)-\beta_{3}(1+m)\\ \ldots \\ \beta_{N+1}(1)-\beta_{N+1}(1+m) \end{array}|\ldots |\begin{array}{c} \beta_{2}(n)-\beta_{2}(n+m)\\ \beta_{3}(n)-\beta_{3}(n+m)\\ \ldots \\ \beta_{N+1}(n)-\beta_{N+1}(n+m) \end{array}].$$ (26)

In Eq. (26), n+m must be less than or equal to the maximum number of depth pixels in the A-scan. The matrix B has N rows and n=p−m columns. Thus, increasing m will reduce the number of columns of matrix B, but it does not change the number of rows of B. The Chebyshev polynomials $\beta_{k}(z)$ are now specified in pixel units as $\beta_{k}(1),\beta_{k}(2),\ldots ,\beta_{k}(n)$, where k=2,3,…,(N+1) and we have simply reassigned the n discrete, evenly spaced values of $z=z_{1},z_{2},\ldots ,z_{n}$ to i=1,2,…,n.

Only two vertically shifted A-scans at the same lateral position, $I_{1}(z)$ and $I_{2}(z)$, are needed to form the row vector y and the matrix B, from which c is computed. From the coefficients in c, the estimated g(z), denoted in pixel space as g^(i), can be computed as

10 log10 g^(i)=c2β2(i)+c3β3(i)+…+cN+1βN+1(i). (27)

Note that because $c_{1}$ cannot be determined by the least-squares operation, the combined function g(z) can only be estimated up to a proportional constant. Although not mathematically necessary, for scaling purposes, we may add a constant to the right-hand-side Eq. (27) so that the maximum of the recovered g^(z) is one. The maximum of g^(z) being one means that the maximum of 10 log10 g^(z) equals zero. The algorithm is summarized below.

**Algorithm 1:** Basis fit.

**Inputs:** Intensity A-scans 1 and 2 in dB scale, vertical shift m in pixels
**Output:** Estimated combined confocal and fall-off function g^(z)
**Step 1:** Choose a number of basis functions N and generate N Chebyshev polynomials from $\beta_{2}(i),\beta_{3}(i),\ldots ,\beta_{N+1}(i)$, where i=1,2,…,n.
**Step 2:** Build y and B according to Eqs. (25) and (26).
**Step 3:** Solve for c by Eq. (24).
**Step 4:** Use the coefficients from c to compute 10 log10 g^(i) by Eq. (27).
**Step 5:** Normalize g^(i) by adding a constant to 10 log10 g^(i) so that its maximum becomes one.
**Step 6:** Convert the result in Step 5 to regular, non-dB scale to obtain g^(i) as follows: g^(i)=10x(i)/10, where x(i)=10 log10 g^(i). If desired, g^(i) can be converted back to depth space g^(z) by reindexing i from 1 to n to z from 0 to $z_{\max}$.

The confocal function varies with the refractive index, thus also impacting the extracted g^(z) for a particular sample being measured. The analytical expression for the confocal function, as assumed by Ratio Fit, depends on two parameters $z_{0}$, the focal plane depth, and $z_{R}$, the apparent Rayleigh range, both of which are dependent on the refractive index of the sample. For an inhomogeneous sample, the shape of the confocal function is stretched and/or compressed as the refractive index varies with depth. Ratio Fit assumes a symmetric form of the confocal function that will not be correct for inhomogeneous samples, but this asymmetry may be negligible depending on the range of refractive indices in the sample. However, Basis Fit does not experience this issue because it builds g(z) from a set of basis functions regardless of how the refractive index compresses or stretches the confocal function. In the experimental results of this paper, the extracted g^(z) is sample-specific and used to correct the same sample. In practice, we often find g(z) for a representative sample and use it to correct the B-scans of similar samples, under the assumption that g(z) does not change significantly across similar samples for the same OCT system.

### 3.3 A-scan Correction

After g^(z) is found, the corrected A-scans are computed as

A^1(z)=I1(z)g^(z), (28)

and

A^2(z)=I2(z)g^(z). (29)

The difference between 10 log10A^1(z) and 10 log10 A^2(z+Δz) is ε(z). Thus, by minimizing the model-to-data fit, we are also minimizing the difference between the corrected, shifted A-scans.

### 3.4 Checking for Ill-conditioning of B

For correct estimation of c, the matrix B must be well-conditioned (i.e., its rank is equal to the number of parameters being solved). The condition number of a matrix is defined as a ratio of its largest to smallest singular value. In practice, if the condition number of a matrix is smaller than $∼6.7\times 10^{8}$, then according to the Institute of Electrical and Electronics Engineers (IEEE) standards for double precision calculation, the matrix is considered well-conditioned.^14^ Above this value, the matrix is considered ill-conditioned. When B is ill-conditioned, the solution C is not unique, leading to incorrect estimation of g(z). Therefore, the condition number of B, which can reveal when B is ill-conditioned, should be monitored.

If ill-conditioning occurs, it can be fixed by reducing the number of basis functions (reducing the number of rows of B) or by involving more vertically shifted A-scans at the same lateral position of the sample (i.e., by adding more columns of B). Reducing the number of basis functions will decrease the rank requirement of B. Increasing the number of A-scans at different depths will increase the rank of B. Note that the former is more practical, as the latter requires that additional images be captured.

In general, as the number of basis functions increases, the estimation of g(z) will improve until ill-conditioning begins to take effect. Ill-conditioning causes the shape of g^(z) to change drastically. Therefore, one way to determine the appropriate number of basis functions is by monitoring the change in the shape of g^(z) before the effects of ill-conditioning can be observed. For the experimental B-scans in this paper, we found that anywhere between 5 and 20 basis functions is sufficient. The vertical shift Δz also impacts the “optimal” number of basis functions. Generally, with a smaller Δz, more basis functions can be used.

### 3.5 Using More Than Two A-scans at Different Depths

If additional vertically shifted A-scans at the same lateral position are available, the derivation to perform Basis Fit can be easily extended. In the above derivation, the sample is shifted only once, producing one pair of A-scans. Suppose now the sample is vertically shifted again to a different depth, producing a third A-scan. Two vertical shifts result in three pairs of A-scans: 1 and 2, 1 and 3, 2 and 3. The derivation can be applied to each pair, and their resulting equations can be combined to find the coefficients c as

$$c=[y_{12}|y_{13}|y_{23}][B_{12}|B_{13}|B_{23}{]}^{+}.$$ (30)

Although the theory allows for more than one vertical shift of a sample (corresponding to two different depths), in practice, we find that one vertical shift is sufficient to recover g(z). However, if several additional vertical shifts are used, and thus increasing the number of columns of B, performing least squares to determine c does not become computationally infeasible. Each submatrix $B_{12},B_{13},B_{23},\ldots$ has N rows and n=p−m columns, where m may be different for each submatrix depending on the vertical shift between the corresponding pair of A-scans. For example, going from one vertical shift (two A-scans) to two vertical shifts (three A-scans) causes the column dimension of B in Eq. (30) to be approximately tripled. In a typical application, this increase in the dimensions is not large enough to create any computational issue when performing linear least squares, which is a relatively simple operation.

### 3.6 Using More Than Two A-scans at Different Lateral Positions

The general theory of Basis Fit applies to a single pair of vertically shifted A-scans at the same lateral position. However, it can be similarly extended to include A-scans from different lateral positions, such as for multiple A-scans in a B-scan. Several algorithms for AC extraction often rely on the use of an averaged A-scan spanning multiple lateral positions. Although not necessary for Basis Fit, such averaging is advantageous because it helps reduce speckle noise. For example, for two vertically shifted B-scans, the original derivation can be applied to the pair of A-scans at each lateral position, to produce $y_{1},y_{2},\ldots$ and $B_{1},B_{2},\ldots$, where the subscript denotes lateral position. The coefficients of c can be solved from

$$c=[y_{1}|y_{2}|\ldots ][B_{1}|B_{2}|\ldots {]}^{+}.$$ (31)

Due to the fact that $B_{1},B_{2},\ldots$, are only dependent on the selected basis functions and the vertical sample shift Δz, all the B matrices are in fact the same, $B_{1}=B_{2}=\ldots =B$. Thus, the solution in Eq. (31) becomes identical to

$$c=\overset{¯}{y}B^{+},$$ (32)

where $\overset{¯}{y}$ is the pixel-wise average of $y_{1},y_{2},\ldots$. The derivation for two B-scans can be similarly extended to involve more than two B-scans, as described in Eq. (30).

### 3.7 Laterally Dependent g^(z)

Although it is possible to use all data from a single B-scan to extract a single g^(z) for the entire B-scan, it is also possible to use small sets of regional data to find a local, laterally dependent g^(z) across the B-scan, which may be useful to better consider sample-dependent effects on the value of g^(z) or lateral differences in the confocal or fall-off functions due to lens aberrations. A moving window consisting of a small number of A-scans across the B-scan can be used to help reduce noise during extraction. For most of the results in this paper, we extract a single g^(z) for the entire B-scan.

### 3.8 Performance Metric

Recall that the mathematical goal of the problem for two A-scans (or two B-scans) is to determine the g(z) that, when removed, yields two corrected A-scans (or B-scans) that become vertically shifted versions of each other by Δz. Therefore, we use the similarity of the corrected A-scans (or B-scans) as a performance metric. To compare the degree to which any two A-scans are similar to each other, we use the root-mean-square error (RMSE) defined for two uncorrected, vertically shifted A-scans $I_{1}(z)$ and $I_{2}(z)$ where z is depth. In pixel units, the RMSE for the uncorrected A-scans is

$$\mathrm{RMSE}=\sqrt{\frac{\sum_{i=1}^{n-m}(I_{1}(i{)}_{\mathrm{dB}}-I_{2}(i+m{)}_{\mathrm{dB}}{)}^{2}}{n-m}},$$ (33)

where $I_{1}(z)$ and $I_{2}(z)$ are specified as $I_{1}(i)$ and $I_{2}(i)$, respectively, for i=1,2,…,n. The variables $I_{1}(i)$ and $I_{2}(i)$ can be replaced by A^1(i) and A^2(i) to quantify the improvement of the RMSE of the corrected A-scans

RMSE=∑i=1n−m(A^1(i)dB−A^2(i+m)dB)2n−m. (34)

The denominator n−m refers to the number of overlapping depth pixels in the two aligned A-scans, and thus, the non-overlapping pixels are removed from the calculation. Note that m is given in pixel units. When the metric is applied to two B-scans, the metric is computed for every corresponding pair of A-scans at each lateral position of the two B-scans.

## 4 Methods

To test the effects of various parameters on the performance of Basis Fit, we performed a number of simulations. Table 1 provides an overview of the simulation conditions described in the following sections. The base sample for all simulations was a B-scan comprising three sample layers extending laterally, each having different refractive indexes and ACs.

**Table 1:** Simulations.

Simulation	Name	Description	Results
A	Model	Noise-free and noise-added three-layer B-scan with confocal and fall-off effects	Computes RMSE for Basis Fit and Ratio Fit
B	Ill-conditioning	Simulation A noise-free sample; vary N, the number of basis functions used to compute g(z).	Demonstrates the effects of ill-conditioning on g(z) and how to correct for ill-conditioning
C	Modified fall-off function	Simulation A noise-free sample with different amounts of fall-off applied (including no fall-off)	Shows insensitivity of Basis Fit to the amount of fall-off
D	Modified confocal function	Simulation A noise-free sample with modified confocal function (without fall-off)	Shows insensitivity of Basis Fit to shape of confocal function
E	AC Estimation	Simulation A noise-added sample	Estimates AC values

### 4.1 Simulation A Design: Model

To validate Basis Fit, our base simulation comprises two B-scans (composed of 200 lateral A-scans each) of a three-layer sample whose intensity diminishes to zero before the end of the imaging range. The ideal, noise-free A-scan at each lateral position of the B-scan took the form

$$A_{1}(z)=k\alpha \beta L_{0}\mu (z)e^{-2\int_{0}^{z}\mu (s)ds},$$ (35)

where α is the fraction of attenuated light that is backscattered, β is the quantum efficiency of the detector, $L_{0}$ is the intensity of the light source, μ(z) is the AC at depth z, and s is a variable of integration.^11^ The variable k was added as a measurement scaling factor. In this simulation, we arbitrarily used α=0.75, β=0.75, $L_{0}=5\;\mathrm{mW}/{\mathrm{mm}}^{2}$, and k=1000. For this simulation, the depth z was given in mm from 0 to 2.5 mm (in pixel units from 1 to 512, corresponding to a 512-pixel A-scan). Each layer was assumed to have a constant AC: the arbitrarily selected ACs were $1\;{\mathrm{mm}}^{-1}$ from 0.073 to 0.561 mm, $2\;{\mathrm{mm}}^{-1}$ from to 0.561 to 1.050 mm, and $4\;{\mathrm{mm}}^{-1}$ from 1.050 to 2.5 mm. This first ideal, noise-free A-scan, denoted $A_{1}(z)$, was then vertically shifted in depth to generate a second noise-free A-scan, $A_{2}(z)$

$$A_{2}(z)=A_{1}(z-\Delta z),$$ (36)

where Δz is the vertical shift in depth, which was 0.2441 mm (m=50  pixels) in this simulation.

To simulate a more realistic A-scan, we set the signal lower limit to −30  dB and then independently added speckle and shot noise to the amplitude (i.e., the square-root of intensity) of the ideal A-scans. The noise-free $a_{i}(z)$ becomes $a_{i}(z)n_{sp}(z)+n_{sh}(z)$, where $a_{i}(z)=\sqrt{A_{i}(z)}$ and i=1,2 for the two A-scans. The multiplicative speckle noise model we used is

$$n_{sp}(z)=\sqrt{-(\frac{4}{\pi })\;\ln(u.r.n(z))},$$ (37)

where u.r.n(z) is a uniformly distributed random number between 0 and 1. The additive shot noise model we used is

$$n_{sh}(z)=\sigma \sqrt{\frac{4\;\ln(u.r.n(z))}{\pi -4}},$$ (38)

where u.r.n(z) is another uniformly distributed random number between 0 and 1, and $\sigma =C\times 10^{-(\frac{\mathrm{SNR}}{20})}$ is the standard deviation of the shot noise term. Due to the fact that μ(z) is depth-dependent, we chose C to be the square root of the average value of the intensity over the entire depth range, $k\alpha \beta L_{0\;}\mu (z)$. SNR is the signal-to-noise ratio in decibels. A SNR of 80 dB was used in this simulation so that visually the noise does not overwhelm the signal. After noise was added to the amplitudes, the two signals were then squared to produce the two initial, noise-added intensity A-scans ${\overset{¯}{A}}_{1}(z)$ and ${\overset{¯}{A}}_{2}(z)$.

To yield the “measured” A-scans, the two noise-added A-scans were then multiplied by separate confocal and fall-off functions to produce two uncorrected A-scans

$${\overset{¯}{I}}_{1}(z)=h(z)f(z){\overset{¯}{A}}_{1}(z),$$ (39)

and

$${\overset{¯}{I}}_{2}(z)=h(z)f(z){\overset{¯}{A}}_{2}(z).$$ (40)

The confocal function was modeled as

$$h(z)=(((z-z_{0})/z_{R}{)}^{2}+1{)}^{-1},$$ (41)

where z is depth, $z_{0}$ is the focal plane depth, and $z_{R}$ is the apparent Rayleigh range.^15^ The values $z_{0}=0.7324\;\mathrm{mm}$ (150 pixels) and $z_{R}=0.3662\;\mathrm{mm}$ (75 pixels) were used for this simulation, resulting in a confocal function that dominates the first half of the imaging range, as often observed in experimental results.

The expression we used for the fall-off function in the simulation is

$$f(z)=\mathrm{sinc}(\zeta {)}^{2}\;\exp(-\frac{w^{2}\zeta^{2}}{2\;\ln\;2}),$$ (42)

where $\zeta =(\pi /2)(z/z_{\mathrm{RD}})$ is the depth normalized to the maximum ranging depth, $z_{\mathrm{RD}}=\lambda^{2}/(4\Delta \lambda )$, λ is the central wavelength, Δλ is the wavelength spacing among pixels, and w=δλ/  Δλ, where δλ is the spectrometer’s spectral resolution (defined by full width at half maximum).^12^ The parameters we used to determine its profile are those of a commercial OCT system (Telesto, Thorlabs) with λ=1310  nm, $\Delta \lambda =7.1875\times 10^{-11}\;m$, and $\delta \lambda =1.1055\times 10^{-10}\;m$. Separate noise was generated and added to each lateral A-scan (400 total, consisting of 200 lateral A-scans for each of the two different sample depths). For each of the two sample depths, 200 lateral A-scans were appended side-by-side to produce a “measured” B-scan.

From the two “measured” B-scans, two laterally averaged A-scans were produced. Basis Fit was applied to extract g(z), which was then used to correct the two laterally averaged A-scans. Data from the two laterally averaged A-scans ${\overset{¯}{I}}_{1}(z)$ and ${\overset{¯}{I}}_{2}(z)$ were used to form the row vector y and the matrix B given in Eqs. (20) and (22), where y(z) is given in Eq. (11). The coefficients $c_{2},c_{2},\ldots ,c_{N}$ contained in c were found according to Eq. (24). The simulation was repeated with and without noise. The results were compared with that obtained by Ratio Fit, which assumes the prescribed form of the confocal function that is the same as that used in this simulation and ignores the fall-off function.

### 4.2 Simulation B Design: Ill-conditioning

To assess the effect of ill-conditioning on the quality of Basis Fit recovery, we tested recovery of g(z) as the number of basis functions varied from 20 to 60. Using the same three-layer sample from Simulation A, two noise-free A-scans were generated with m=50  pixels apart. The same confocal and fall-off functions were applied. For this simulation, B became ill-conditioned at N=60 basis functions. To show how the problem of ill-conditioning can be remedied by involving a third A-scan vertically offset at a different depth, we simulated a third A-scan, which was the first A-scan vertically shifted by 80 pixels.

### 4.3 Simulation C Design: Modified Fall-off Function

**Fig. 1:**
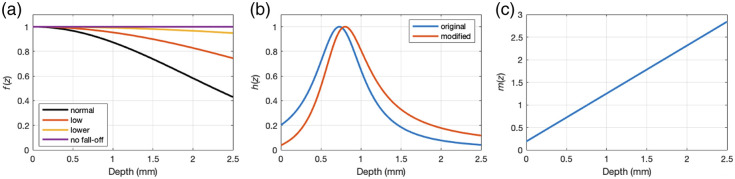
(a) Varying levels of fall-off functions used to generate the simulated intensity profiles for Simulation C. (b) Modified confocal function used to generate the simulated intensity profile for Simulation D. (c) Modification function m(z) used to modify the original confocal function in (b).

To illustrate the effect of fall-off on Basis Fit and Ratio Fit recovery, we used the noise-free three-layer sample from simulation A and considered four scenarios: no fall-off, normal fall-off (derived from the parameters of the Telesto mentioned previously), low fall-off, and lower fall-off. A plot of the fall-off functions used appears in Fig. 1(a). To assess the performance of the algorithms for these conditions, we computed the RMSE of the corrected A-scans for each scenario.

### 4.4 Simulation D Design: Modified Confocal Function

To illustrate the robustness of the basis function method against differences in g(z) from their expected functional forms, we used the noise-free Simulation A sample and applied a new confocal function that was modified from its prescribed function, shown in red in Fig. 1(b). The modified confocal function was generated by multiplying the original confocal function by the modification function m(z), shown in Fig. 1(c), to represent a slight deviation in the assumed form of the confocal function and/or unknown and unmodeled depth-dependent effect. No fall-off was present in this simulation to isolate the effect of the modified confocal function. We then compared the RMSE of the corrected A-scans by Basis Fit and by Ratio Fit.

### 4.5 Simulation E Design: AC Estimation

Using the noise-added samples from Simulation A, we compared the extracted ACs of each of the three layers by curve fitting (CF)^10^^,^^16^ after correction by Basis Fit and Ratio Fit.

### 4.6 Experiment A design: $\mathrm{PDMS}/{\mathrm{TiO}}_{2}$

To further validate the method, we demonstrated its use on two B-scans of a three-layer polydimethylsiloxane and titanium dioxide ($\mathrm{PDMS}/{\mathrm{TiO}}_{2}$) phantom with the surface positioned at two different depths (Δz=101  μm or m=10  pixels). From top to bottom, the phantom consisted of 0.3, 0.03, and 0.3% weight ${\mathrm{TiO}}_{2}$. The images were taken on a commercial SD-OCT system (Telesto, Thorlabs) with a setting of 20 A-scan averaging. Before extraction of g(z), the B-scans were cleaned and smoothed for debiasing and speckle noise removal by the following procedure described in Dwork et al.^6^ To debias the data, the median of the intensity signal in the air portions above the samples of the two B-scans (in non-dB scale) was used as an estimate of the mean of the additive noise and subtracted off from the intensity at each pixel of the B-scans, unless this value was lower than the minimum value of the B-scans. Once debiased, the effect of speckle was reduced by a 2D Gaussian filter of a standard deviation of 0.5 pixels. This value was chosen to suppress high-frequency noise while maintaining the structure of the signal. We then used a separate rotationally symmetric low-pass Gaussian filter with a size of 10 pixels and a standard deviation of five pixels to smooth the B-scans (in dB-scale). According to Dwork et al.,^6^ high frequencies in the B-scans corrupt the extraction of the confocal function. The parameters of the low-pass Gaussian filter were chosen to reduce this corruption by smoothing the images further. The final AC results were not sensitive to these parameters. The cleaned and smoothed B-scans were used to find g^(z) using Basis Fit and Ratio Fit. For all three experimental datasets, a single g^(z) was extracted for the entire B-scan from two laterally averaged A-scans (converted into dB-scale). After correction of the cleaned and smoothed B-scans by the g^(z) extracted by Basis Fit and Ratio Fit, we used the CF method to compute the AC values.

Ratio Fit assumes that fall-off is either negligible or removed beforehand. For measurements made on our Telesto (corresponding to Experiments A and B), a fall-off curve was measured by movement of the reference arm to image a mirror sample at different depths. The acquired fall-off was removed from the B-scans before applying Ratio Fit to find the confocal function. Thus, g(z) for Ratio Fit was found by multiplying the extracted confocal function with the measured fall-off function. Note that when the fall-off was removed, the data that the models for the two methods tried to fit were different. Basis Fit uses the cleaned and smoothed B-scans to find the combined confocal and fall-off function, whereas Ratio Fit uses the cleaned and smoothed B-scans after correction by fall-off to find only the confocal function.

### 4.7 Experiment B Design: Cucumber Slice

To demonstrate this method on biological data, we collected two B-scans of a cucumber slice on the Telesto with a setting of 20 A-scan averaging at two different depths (Δz=206  μm or m=41  pixels) and performed the same analysis. The B-scans were cleaned by the same procedure explained in Sec. 4.6 and smoothed. For less homogeneous samples, stronger smoothing (by a wider low-pass Gaussian filter than for the $\mathrm{PDMS}/{\mathrm{TiO}}_{2}$ phantom) was used to decrease the effects of noise in the intensity signals so that g^(z) does not model the noise in the data. The same measured fall-off function obtained in Experiment A for the Telesto was removed for the B-scans of this experiment before applying Ratio Fit to find the confocal function.

### 4.8 Experiment C Design: Human Retina

We also tested this method on two B-scans of an in-vivo normal human retina collected at two different distances (Δz=535  μm or m=274  pixels) from the zero pathlength delay. The images were taken with a clinical SD-OCT system ($\lambda_{0}=840\;\mathrm{nm}$, Cirrus high-definition OCT model 5000, Carl Zeiss) with a lateral and axial resolution in tissue of 15 and 5  μm, respectively. The data and code to process the B-scans came from a publicly available dataset;^17^ hence, no approval from the Institutional Review Board (IRB) was required for this secondary analysis. The B-scans were cleaned by the same procedure explained in Sec. 4.6 and smoothed with the same low-pass Gaussian filter used in Sec. 4.7. Fall-off correction was not applied to Ratio Fit for the eye data because the fall-off parameters for this OCT system were not available. The function g(z) was then extracted from these two processed B-scan images and used to generate the corrected B-scan images from the cleaned B-scans. In addition, Basis Fit was repeatedly applied to different lateral positions of the B-scans comprising windowed regions of 25 lateral A-scans each, producing a series of laterally dependent g(z). This result was compared with when a single g(z) was extracted for the entire B-scan. Finally, we investigated the impact of the improved g^(z) by Basis Fit on the estimated AC values. Basis Fit does not assume a single-scattering model in finding g(z), but after g(z) is found, the determination of the AC depends on whether a single or multiple-scattering model is assumed. In this paper, we use the CF and depth-resolved confocal (DRC)^4^^,^^11^ methods, both of which assume a single scattering model. For example, in the CF method, the AC is extracted from the slope of the natural log of the intensity versus depth from the corrected A-scan. If instead a multiple scattering model is used, then some analytical form of the intensity versus depth is assumed, and a nonlinear search is used to find the AC. A multiple scattering model could provide more accurate AC results but is often too complicated for implementation.^10^

## 5 Results and Discussion

### 5.1 Simulation A results: Model

**Fig. 2:**
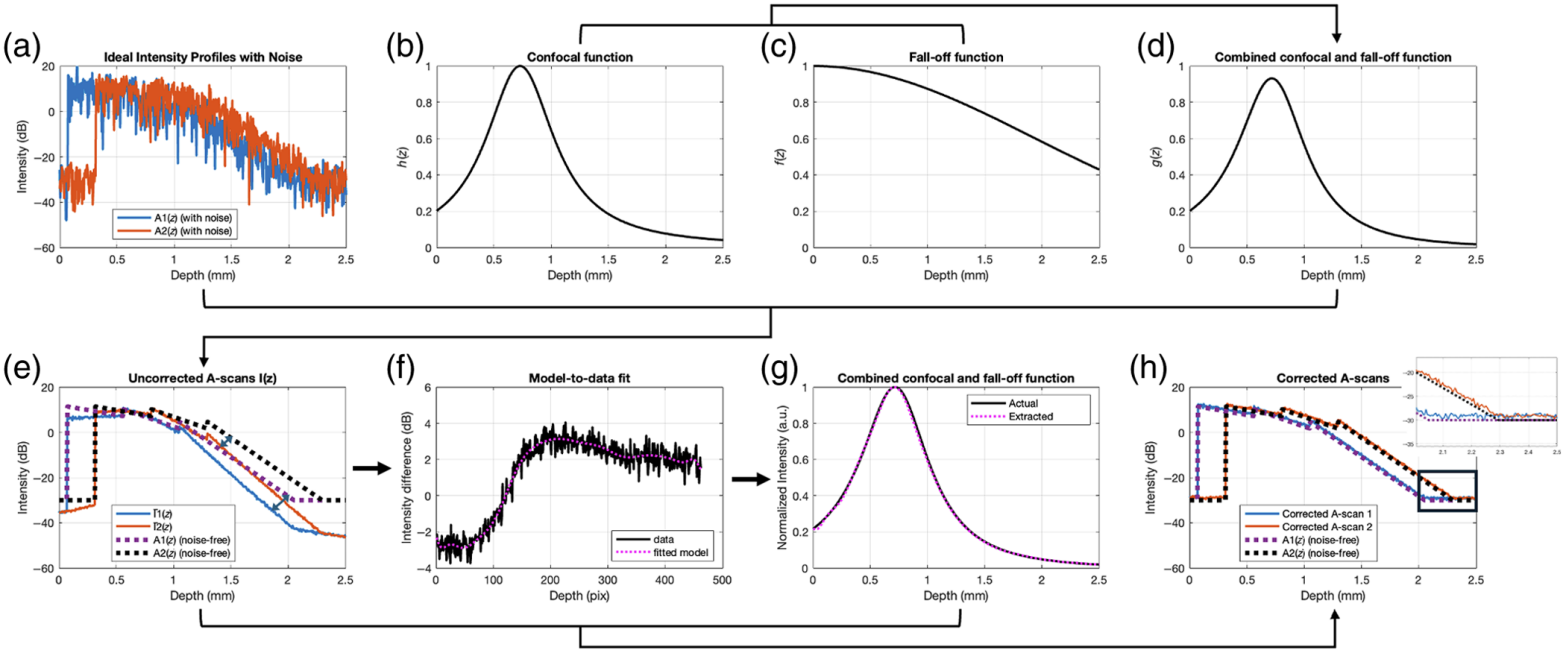
Results of simulation with N=20 basis functions. (a) original ideal A-scans with noise at a single lateral position from the B-scans, (b) simulated confocal function, (c) simulated fall-off function, (d) combined simulated confocal and fall-off function, and (e) uncorrected laterally averaged A-scans (solid) with original noise-free A-scans (dotted) overlayed. The arrows highlight where the original and uncorrected A-scans differ due to g(z). (f) Model-to-data fit, (g) estimated g(z), and (h) corrected laterally averaged A-scans (solid) with original noise-free A-scans (dotted) overlayed. The actual confocal function (scaled so that its maximum is one) and true A-scans are shown for comparison in panels (g)–(h). The zoomed inset of panel (h) highlights the small discrepancy between the true and corrected A-scans due to noise in low signal regions. The arrows in panel (e) indicate the deviation of the uncorrected A-scans from the ideal A-scans.

Figure 2 shows the successful extraction of g(z) and recovery of the two ideal A-scans. Figures 2(a)–2(d) show the noise-added simulated ideal A-scans (with speckle noise and shot noise at a single lateral position), confocal function, fall-off function, and combined function used to generate the laterally averaged uncorrected A-scans (solid lines) in Fig. 2(e), which are overlayed on the two original (noise-free and confocal and fall-off free) A-scans (dotted lines). Discrepancies between the two [ideal A(z) and measured A-scans I(z)] are marked with arrows and manifest as a strong skew from the baseline starting about 1 mm from the surface of each A-scan. The discrepancies are caused by confocal and fall-off effects. The arrows point to regions where the noise-free $A_{1}(z)$ and $A_{2}(z)$ (dotted lines) do not match with the measured ${\overset{¯}{I}}_{1}(z)$ and ${\overset{¯}{I}}_{2}(z)$ (solid lines), respectively, due to g(z). Using 20 Chebyshev basis functions, the model-to-data fit, plotted as a comparison of Y (data) and cB (model) versus depth, is shown in Fig. 2(f), and the resulting recovered g^(z) in Fig. 2(g) shows good agreement with the expected function. In Fig. 2(g), this combined function is normalized so that its maximum is one. Finally, the combined function was used to correct A-scans $I_{1}(z)$ and $I_{2}(z)$ to produce the two corrected A-scans (solid lines) shown in Fig. 2(h), which were then overlayed on the two original A-scans (dotted lines). Note that in a simulation with no noise, the overlays in Figs. 2(g) and 2(h) are practically indistinguishable (not shown); the small discrepancy between the true and corrected A-scans is due to the noise from the very low signal in that region of the A-scan (<−30  dB).

Table 2 evaluates the performance of the two methods as a function of different noise conditions using the metric of performance given by Eq. (34). As illustrated in the first row of Table 2, when fall-off is present, increasing the number of basis functions improves the estimation of g(z) in the noiseless condition. Importantly, this Basis Fit performs better than the Ratio Fit, both with and without shot noise. Ratio Fit uses the same prescribed form of the confocal function as that used by the simulation; however, as Ratio Fit neglects fall-off, it fails to capture its effects during extraction of the confocal function, which likely accounts for its worse performance here when less noise is present. Note that the RMSE is in units of dB, and the bolded numbers represent the best result of each row.

**Table 2:** Simulation A: RMSE (dB) of the two corrected A-scans by Basis Fit for a different number of basis functions in comparison to Ratio Fit.

	Basis Fit	Basis Fit	Basis Fit	Basis Fit	Ratio Fit
	N=5	N=10	N=15	N=20	
No noise	0.441	0.0726	0.0086	$7.60\times 10^{-4}$	0.389
Only shot noise (80 dB)	0.441	0.0729	0.0106	**0.0065**	0.389
Speckle and shot noise (80 dB)	0.640	0.462	0.450	**0.448**	0.579

### 5.2 Simulation B results: Ill-conditioning

**Fig. 3:**
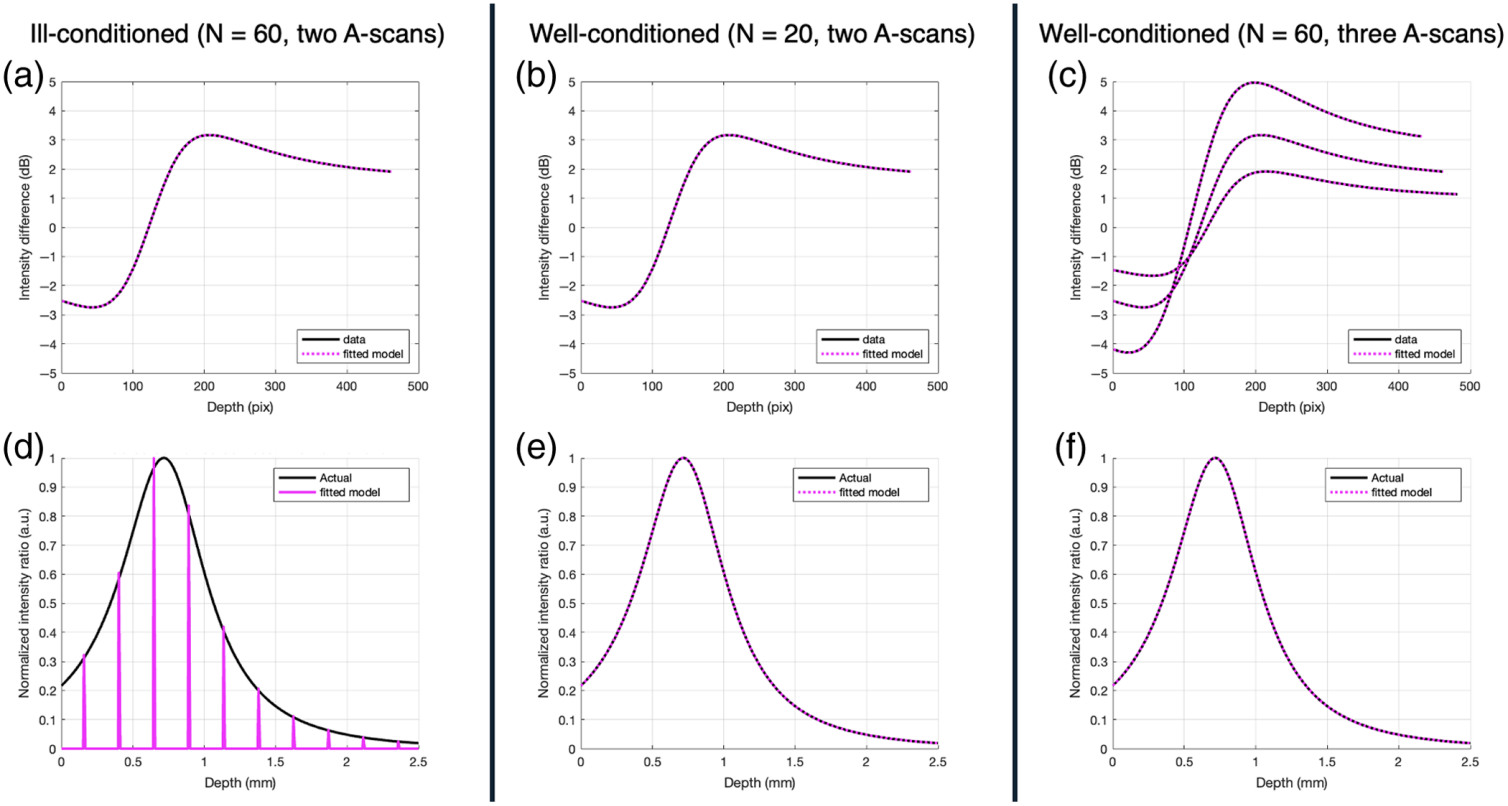
Results of a simulation of ill-conditioning and how to correct it. Panel (a) is the model-to-data fit with ill-conditioning. Panels (b) and (c) are the model-to-data fits with correction by decreasing the number of basis functions or by increasing the number of vertically shifted A-scans at the same lateral position, respectively. Panel (d) is the incorrect recovered combined function (N=60, two A-scans of two depths). Panels (e) and (f) are the correct combined functions by decreasing the number of basis functions (N=20, two A-scan of two depths) or by increasing the number of vertically shifted A-scans at the same lateral position (N=60, three A-scans of three depths), respectively. Note the model-to-data fit plots are in log scale.

Figure 3 shows the effect of conditioning on accurate recovery of the combined function and the result of various methods to correct for ill-conditioning by plotting the model-to-data fits in a log scale. The three sets of model-to-data fits shown in Figs. 3(a)–3(c) produce the three g^(z) functions in Figs. 3(d)–3(f); the three sets of model-to-data curves in Fig. 3(c) correspond to the individual fits for the three pairs of A-scans: $A_{1}(z)$ and $A_{2}(z)$, $A_{1}(z)$ and $A_{3}(z)$, and $A_{2}(z)$ and $A_{3}(z)$ are shown separately for convenience (in the algorithm, the three fits are concatenated into a single matrix). Note that the model-to-data fit plots are in log scale and show the difference between the two A-scans for the model and data, as explained below in Eq. (24). In all cases, the fit of the model-to-data shows a practically perfect overlap between the data and the fitted model, as shown in Figs. 3(a)–3(c). When a large number of basis functions is used (N=60), the matrix B in Eq. (19) becomes ill-conditioned (condition number of $B:\;8.8541\times 10^{12}$). In this case, the recovered g^(z) is incorrect, as shown in Fig. 3(d): although some of the points for the recovered function roughly match the curve for the actual function, many are zeros. This situation can be corrected by reducing the number of basis functions from N=60 to 20, as shown in Fig. 3(e) (condition number of B:  9.7020) or by increasing the number of vertically shifted A-scans at the same lateral position to three, as shown in Fig. 3(f) (condition number B:  9.0641). The correction is seen in that the extracted g^(z) more closely matching the simulated combined function.

### 5.3 Simulation C Results: Modified Fall-off Function

Table 3 shows that Basis Fit consistently captures g^(z) well, regardless of the amount of fall-off, whereas Ratio Fit (ignores fall-off) performs well only when the fall-off function is negligible. In general, Ratio Fit improves as the fall-off effect is reduced. In this simulation, because Ratio Fit knows the form of the simulation confocal function perfectly, it found the confocal function perfectly when there is no fall-off. The bolded numbers in the table represent the best result for each row.

**Table 3:** Simulation C: Comparison of RMSE (dB) of corrected A-scans by basis functions method and Ratio Fit method with varying levels of fall-off.

	Basis Fit (N=30)	Ratio Fit
Normal fall-off	$1.33\times 10^{-5}$	0.389
Low fall-off	$1.33\times 10^{-5}$	0.137
Lower fall-off	$1.33\times 10^{-5}$	0.0253
No fall-off	$1.33\times 10^{-5}$	$4.109\times 10^{-15}$

### 5.4 Simulation D results: Modified Confocal Function

Table 4 shows that Basis Fit consistently captured g(z) correctly, even when the actual confocal function did not perfectly match with the standard form defined by $z_{0}$ and $z_{R}$. By contrast, Ratio Fit performed worse when the standard form of the confocal function did not match with the actual confocal function present in the data. The bolded numbers in the table represent the best result for each row.

**Table 4:** Simulation D: Comparison of RMSE (dB) of corrected A-scans by basis functions method and Ratio Fit method with a modified confocal function.

	Basis Fit (N=30)	Ratio Fit
Original confocal function	$1.33\times 10^{-5}$	$4.109\times 10^{-15}$
Modified confocal function	$1.33\times 10^{-5}$	1.16

### 5.5 Simulation E Results: AC Estimation

From the corrected laterally averaged A-scans with both speckle and shot noise by 20 basis functions in Simulation A, the AC values were computed. These AC values are from the corrected laterally averaged A-scans obtained from the B-scan with a higher sample position. Table 5 shows the improvement in the estimated AC values of the three layers after correction by Basis Fit (bolded) over Ratio Fit. Although the Ratio Fit method ignores fall-off (leading to even more error as one goes deeper into the simulated sample), the form of the confocal function it assumes is identical to that used to produce the simulation data. Note that failure to correct for fall-off leads to a consistent overestimation of the AC by Ratio Fit.

**Table 5:** Simulation E: Comparison of AC of simulation three-layer phantom.

	AC layer 1 (${\mathrm{mm}}^{-1}$)	AC layer 2 (${\mathrm{mm}}^{-1}$)	AC layer 3 (${\mathrm{mm}}^{-1}$)
Exact Values	1	2	4
Basis Fit (N=20)	**0.988**	**2.010**	**4.007**
Ratio Fit	1.075	2.070	4.176

### 5.6 Experiment A Results: PDMS/TiO2

**Fig. 4:**
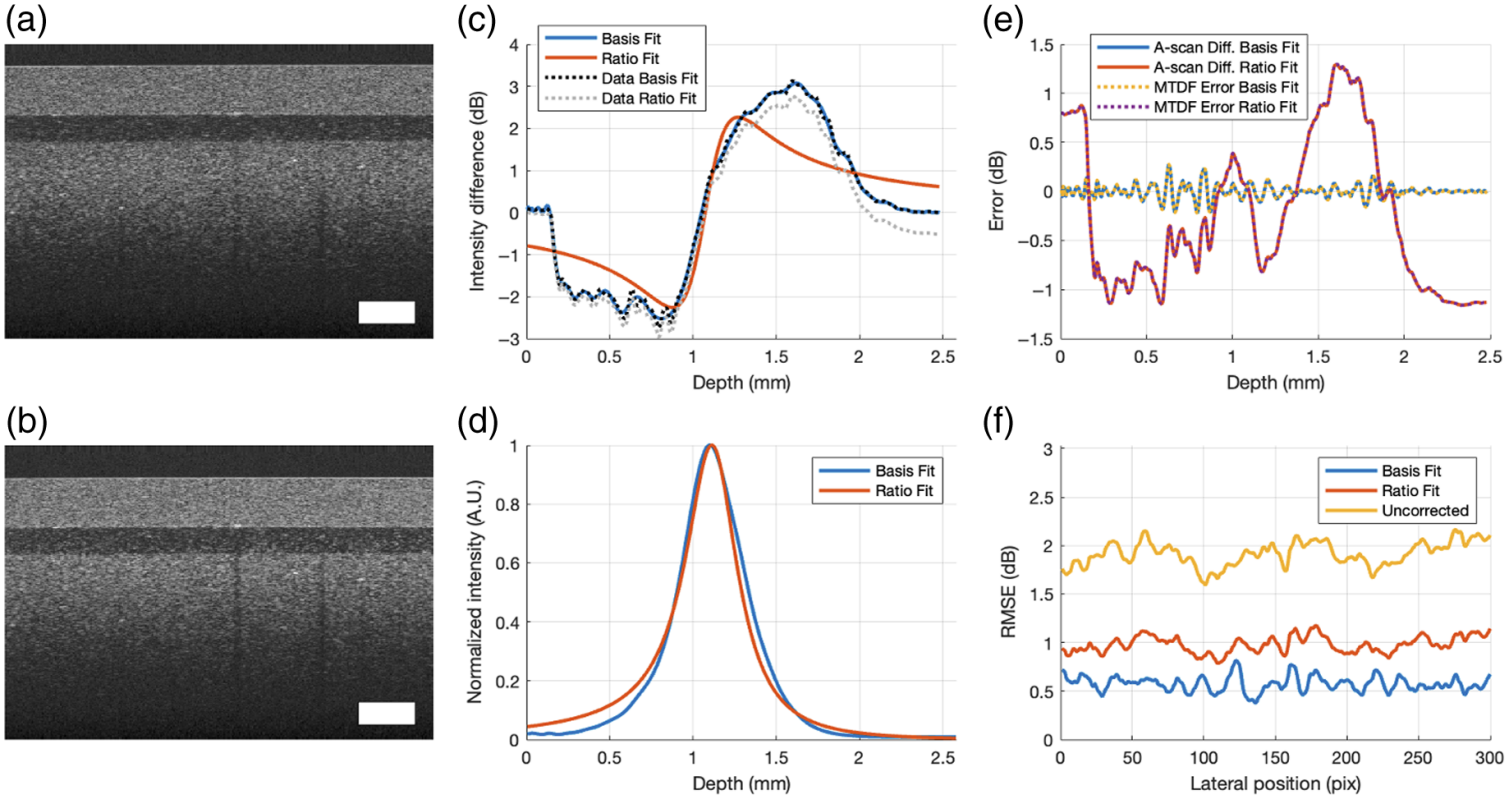
Results of a $\mathrm{PDMS}/{\mathrm{TiO}}_{2}$ phantom. Panels (a) and (b) are the input B-scans. The white rectangle represents 0.2×0.2  mm. Panel (c) is the data-to-model fit by Basis Fit and Ratio Fit. Panel (d) is the extracted g(z) by Basis Fit (N=50) and Ratio Fit. Panel (e) is a comparison of the model-to-data fit error and the difference between the two corrected laterally averaged A-scans by Basis Fit and Ratio Fit. Panel (f) is the RMSE of the two corrected B-scans at each lateral position. The white rectangle and all the axes assume a refractive index of 1.

Figures 4(a) and 4(b) show the two vertically shifted B-scans of the PDMS phantom. These B-scans represent the original processed data prior to any data cleaning. The model-to-data fit by Basis Fit and Ratio Fit in Fig. 4(c) obtained from the cleaned and smoothed B-scans were used to extract the corresponding g^(z)’s shown in Fig. 4(d). Generally, increasing the number of basis functions allows for greater degrees of freedom leading to a better model-to-data fit to extract a more accurate g^(z) using Basis Fit. However, as shown in a previous simulation, too many basis functions lead to ill-conditioning and a poorly extracted g^(z). For this dataset, 50 Chebyshev basis functions were used to avoid the ill-conditioning threshold for this sample. Figure 4(e) shows that the model-to-data fit error, defined as the difference between the cleaned and smoothed data y and the model y^=cB, is identical to the difference between the two laterally averaged corrected A-scans for both methods. To visualize this, we compute the difference in intensity between the two laterally averaged corrected A-scans once they have been cropped and shifted to include the same regions of the sample. This figure confirms that minimizing the model-to-data fit is equivalent to minimizing the difference between the two corrected A-scans (or B-scans) when aligned. The RMSEs of the difference between the two uncorrected and corrected B-scans at each lateral position are shown in Fig. 4(f). The errors and RMSE values are shown in dB in Figs. 4(e) and 4(f) because the model-to-data fit used to extract g^(z) by these methods are also in dB. This figure shows that Basis Fit produces a smaller difference in the corrected images, due to the significantly improved model-to-date fit when compared with the results obtained by Ratio Fit.

**Fig. 5:**
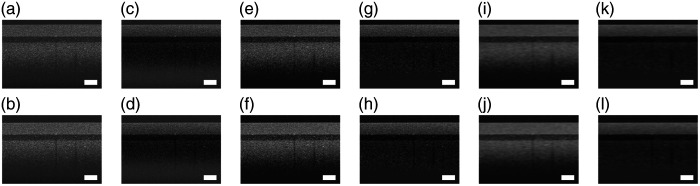
Comparison of uncorrected and corrected B-scans from removal of g^(z) extracted by Basis Fit. Panels (a) and (b) are the raw B-scans, and panels (c) and (d) are their respective corrected B-scans. Panels (e) and (f) are the uncorrected cleaned B-scans, and panels (g) and (h) are their respective corrected B-scans. Panels (i) and (j) are the uncorrected cleaned and smoothed B-scans, and panels (k) and (l) are their respective corrected B-scans. The white rectangle represents 0.2×0.2  mm, assuming a refractive index of 1. All the images have the same color limits for comparison.

Figure 5 shows the B-scans before and after correction by g^(z) extracted by Basis Fit. Although cleaning and smoothing are used to aid in the extraction of g^(z), correction can be done on any of the raw, cleaned, or cleaned and smoothed B-scans. Before correction, g^(z) is rescaled so that the minimum of the air portion (pixels 1 to 100 in depth) of g^(z) is one so that the color limits of the uncorrected and corrected B-scans are more similar to each other for visual comparison. Note that for the rest of the paper, g^(z) is scaled so that its maximum is one.

**Fig. 6:**
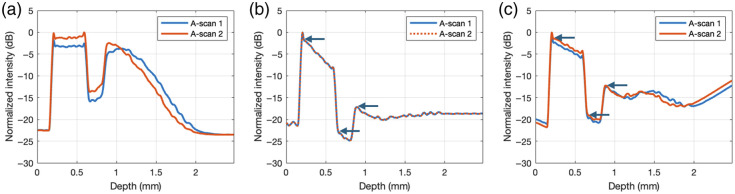
Laterally averaged A-scans of a $\mathrm{PDMS}/{\mathrm{TiO}}_{2}$ phantom before and after correction. Panel (a) is the original laterally averaged A-scans. Panel (b) is the corrected laterally averaged A-scans by Basis Fit. Panel (c) is the corrected laterally averaged A-scans by Ratio Fit. Arrows highlight the start of each layer seen in the corrected A-scans. To show the similarity and difference between the pairs of A-scans before and after correction, the second A-scan in the pair is vertically shifted up by Δz to align with the first A-scan. All the axes assume a refractive index of 1.

To compare how similar the two B-scans are to each other, we computed the averages of the A-scans across all lateral positions for each B-scan and plotted them in Figs. 6(a) and 6(b) before and after correction by Basis Fit, respectively. To help visualize the overlay of the two A-scans for each subplot, the second A-scans are shifted by m pixels to align with the first A-scans. Note that before correction, the laterally averaged A-scans are not merely shifted versions of each other, but after correction, they become shifted versions of each other. Furthermore, it is only after correction that the true attenuation in the sample associated with Beer’s law is revealed as the slope associated with the layers in Fig. 6(b): the arrows show the start of the layers in the corrected A-scans. This attenuation was undetectable in the original uncorrected images in Fig. 6(a) due to the confocal and fall-off effects. Although the corrected laterally averaged A-scans by Ratio Fit in Fig. 6(c) also show linear portions, the overlap between the two A-scans was not as strong as the overlap observed with Basis Fit, and it deviates more at larger depths, where fall-off plays a bigger role.

**Fig. 7:**
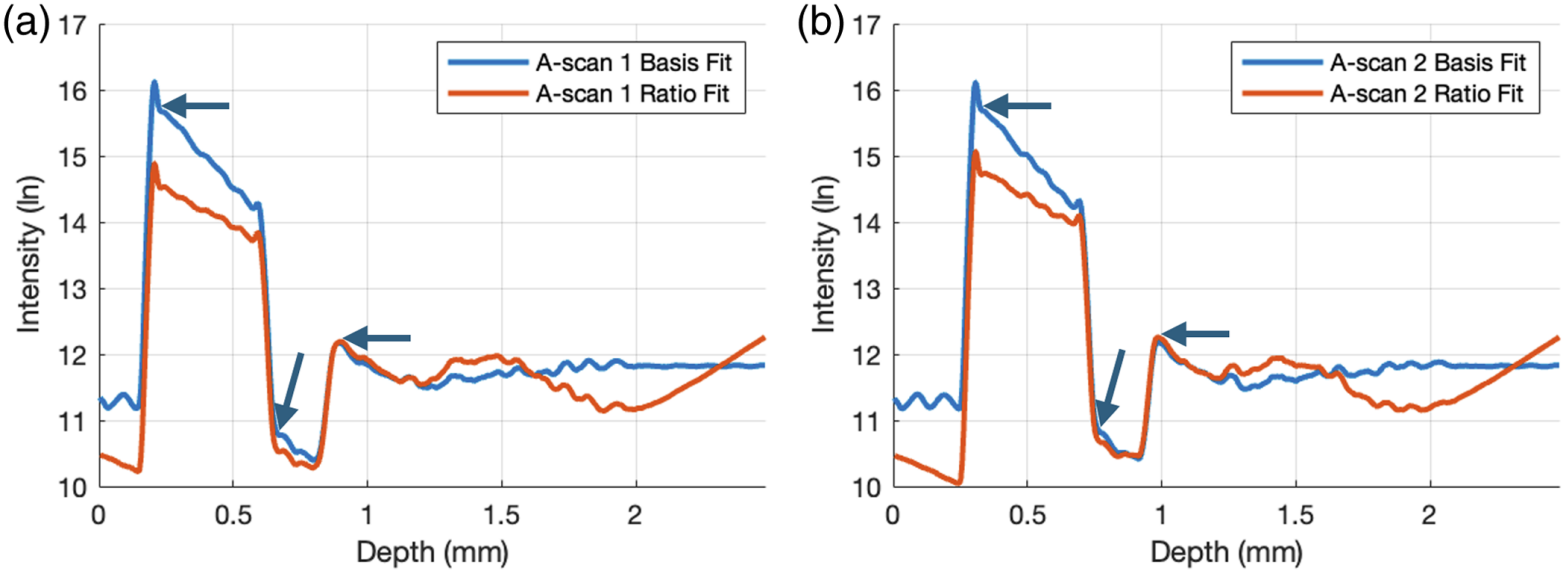
Natural log laterally averaged corrected intensity profiles of $\mathrm{PDMS}/{\mathrm{TiO}}_{2}$ for (a) the first A-scan by both methods and (b) the second A-scan by both methods. Note these are no longer normalized. All the axes assume a refractive index of 1.

Figure 7 shows an overlay of the natural log intensities of the first and second A-scans from Figs. 6(b) and 6(c). To reveal the difference in the results between the two methods, the intensity profiles are no longer normalized. The slope of the intensities in the natural log is equal to $-2\mu_{T}$ where $\mu_{T}$ is the total AC of the region of interest. Because the sample is uniform within each layer (start signified by arrows), the natural log of the signal decays linearly with depth, which is in fact observed here. Visually, it can be seen in Fig. 7 that the slopes of the first layer of the phantom by Basis Fit and Ratio Fit were different, signifying a difference in the AC estimated by the two methods.

Although the extracted g(z) by both methods appeared to be very similar [Fig. 6(b)], correction by Basis Fit and Ratio Fit can lead to different AC values as shown in Table 5. The AC value for the first layer of the phantom composed of 0.3% ${\mathrm{TiO}}_{2}$ in PDMS was measured to be $3.30\;{\mathrm{mm}}^{-1}$ on a spectrophotometer (Cary 7000, Agilent) at the Telesto’s central wavelength of 1310 nm. Following extraction of g^(z) by Basis Fit and Ratio Fit, the A-scans were corrected, and the AC values were computed by the CF method. We only computed the AC values of the first layer (in the interval from 0.25 to 0.55 mm in depth in Fig. 7) because the intensities of the corrected A-scans are near the noise floor for the second and third layers. In addition, it has been shown that the AC obtained by the CF method can be inconsistent for a multi-layer sample, especially for deeper layers and/or layers with smaller concentrations of ${\mathrm{TiO}}_{2}$ (≤0.1 w%).^11^ Compared with Ratio Fit, the estimated AC values by Basis Fit were closer to the measured value by the spectrophotometer for 0.3% ${\mathrm{TiO}}_{2}$ in PDMS for the first layer of the phantom. Because corrected A-scans by Basis Fit were more similar to each other than the corrected A-scans by Ratio Fit, as shown in Figs. 6(b) and 6(c), the AC values computed from each A-scan were also more consistent with each other by Basis Fit than Ratio Fit, as seen by a difference of 0.01 and $0.15\;{\mathrm{mm}}^{-1}$, respectively, in the third column of Table 6. Although the depth in the above figures was given with a refractive index of 1, the refractive index of the first layer of the phantom of 1.46 was used to compute the AC values, as determined by comparing the physical thickness of the sample as measured by micrometer with the optical pathlength visualized with OCT.

**Table 6:** AC (by CF method) of the first layer of the $\mathrm{PDMS}/{\mathrm{TiO}}_{2}$ phantom by Basis Fit and Ratio Fit and difference in the AC values from the two A-scans.

	A-scan 1 (${\mathrm{mm}}^{-1}$)	A-scan 2 (${\mathrm{mm}}^{-1}$)	Difference (${\mathrm{mm}}^{-1}$)
Spectrophotometer	3.30		N/A
CF after Basis Fit	3.18	3.19	0.01
CF after Ratio Fit	1.59	1.74	0.15

### 5.7 Experiment B Results: Cucumber Slice

**Fig. 8:**
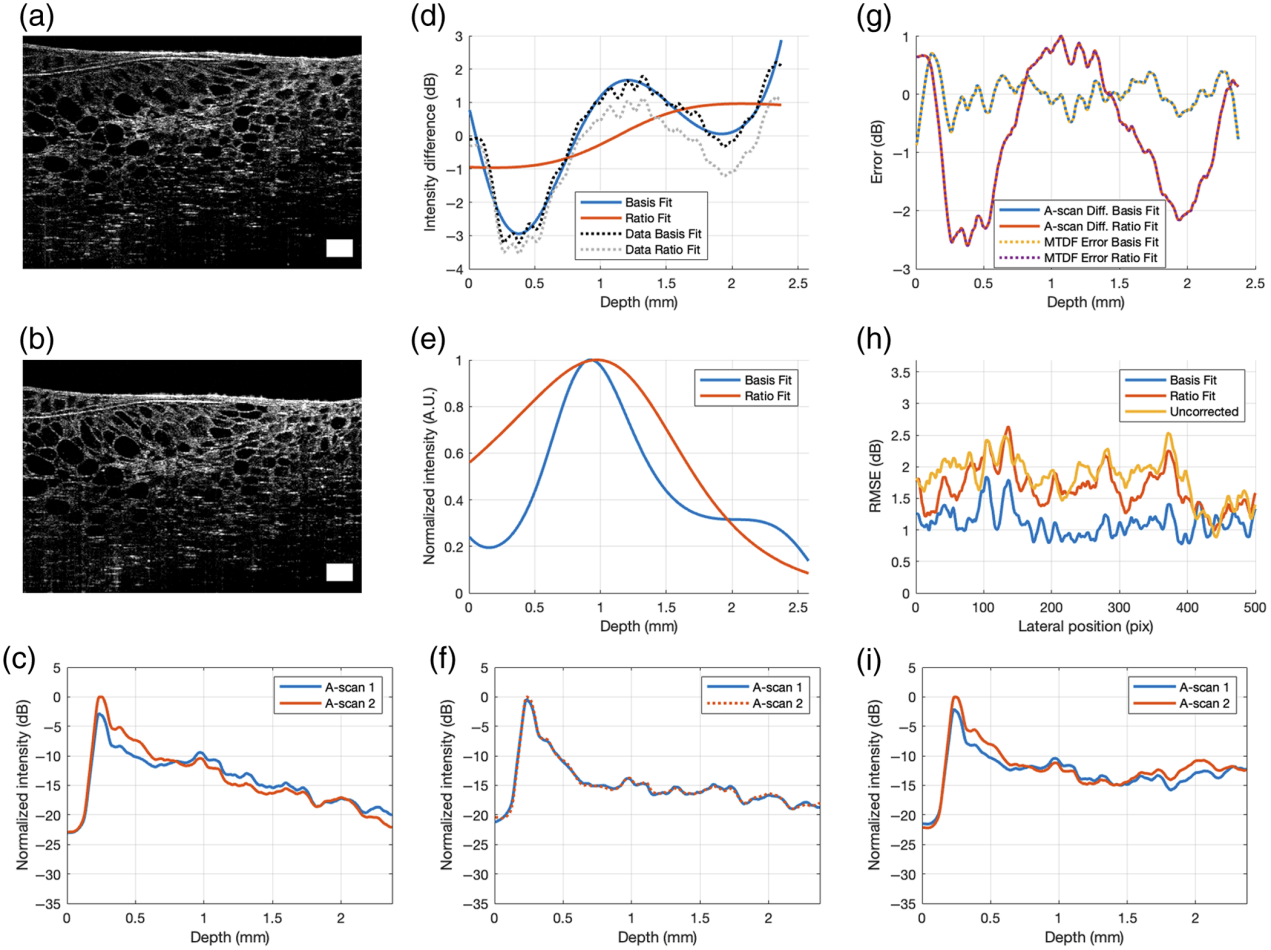
Results of a cucumber slice. Panels (a) and (b) are the input B-scans. The white rectangle represents 0.2×0.2  mm. Panel (d) is the data-to-model fit by Basis Fit and Ratio Fit. Panel (e) is the extracted g(z) by Basis Fit (N=8) and Ratio Fit. Panel (g) is a comparison of the model-to-data fit error and the difference between the two corrected laterally averaged A-scans by Basis Fit and Ratio Fit. Panel (h) is the RMSE of the two corrected B-scans at each lateral position. Panel (c) is the original laterally averaged A-scans. Panel (f) is the corrected laterally averaged A-scans by Basis Fit. Panel (j) is the corrected laterally averaged A-scans by Ratio Fit. To show the similarity and difference between the pairs of A-scans before and after correction, the second A-scan in the pair is vertically shifted up by Δz to align with the first A-scan. The white rectangle and all the axes assume a refractive index of 1.

Figure 8 shows the results for the cucumber slice sample. For this particular non-uniform sample, there was less room for correction because the two original uncorrected B-scans were very similar to each other due to the small vertical shift m in pixel units. Nonetheless, Basis Fit still managed to find a g(z) that improved the similarity between the two corrected images, whereas Ratio Fit provides practically no correction, as evidenced by the poor model-to-data fit of Ratio Fit. Figure 8(g) shows that the difference between the two corrected A-scans after correction of g^(z) by Basis Fit is smaller than that by Ratio Fit. Perfectly overlaid on the solid lines are the dotted lines, confirming that the error in model-to-data fit (dotted lines) is identical to the difference between the two corrected A-scans (solid lines). In addition, Fig. 8(h) shows that the RMSE for all lateral positions of the corrected B-scans is the lowest for Basis Fit. This example shows that Basis Fit is able to produce an improvement over Ratio Fit in extracting g(z) for this biological sample even when the vertical shift is relatively small.

The bottom row of Fig. 8 shows a comparison of the overlap of the two laterally averaged A-scans before and after correction with the extracted g^(z) by Basis Fit and Ratio Fit. For Basis Fit, Fig. 8(f) shows how the two A-scans are shifted versions of one other in intensity. This result cannot be said of Figs. 8(c) and 8(i) before correction and after correction with Ratio Fit.

### 5.8 Experiment C Results: Human Retina

**Fig. 9:**
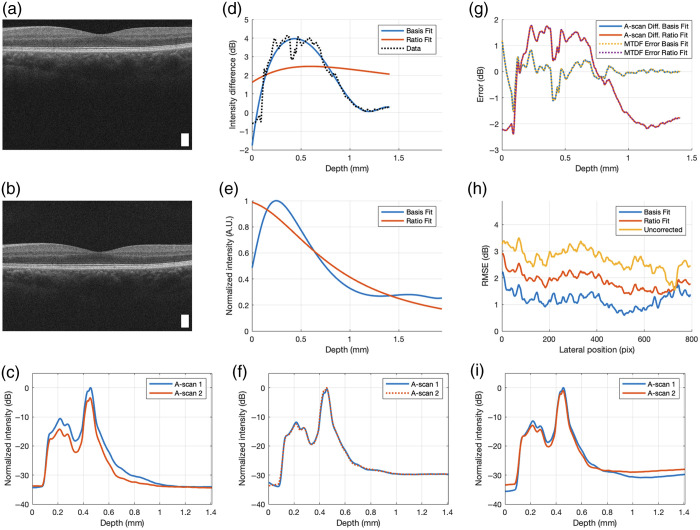
Combined confocal and fall-off function extraction of human retina images. Panels (a) and (b) are the input B-scans. The white rectangle represents 0.2×0.2  mm. Panel (d) is the data-to-model fit by Basis Fit and Ratio Fit. Panel (e) is the extracted g^(z) by Basis Fit (N=7) and Ratio Fit. Panel (g) is a comparison of the model-to-data fit error and the difference between the two corrected laterally averaged A-scans by Basis Fit and Ratio Fit. Panel (h) is the RMSE of the two corrected B-scans at each lateral position. Panel (c) shows the original laterally averaged A-scans. Panel (f) shows the corrected laterally averaged A-scans by Basis Fit. Panel (j) shows the corrected laterally averaged A-scans by Ratio Fit. To show the similarity and difference between the pairs of A-scans before and after correction, the second A-scan in the pair is vertically shifted up by Δz to align with the first A-scan. The white rectangle and all the axes assume a refractive index of 1.

Figure 9 shows the results for the human retina. As shown in Fig. 9(h), g^(z) found by Ratio Fit reduces the difference between the corrected images, but Basis Fit improves it even further. At all lateral positions, the RMSE of the A-scans corrected by g^(z) extracted by Basis Fit is less than the RMSE of the A-scans corrected by g^(z) extracted by Ratio Fit and the RMSE of the uncorrected A-scans. Although the two B-scans in Figs. 9(a) and 9(b) visually appear to be very similar, Basis Fit was able to extract a g^(z) that was an improvement over the g^(z) extracted by Ratio Fit.

**Fig. 10:**
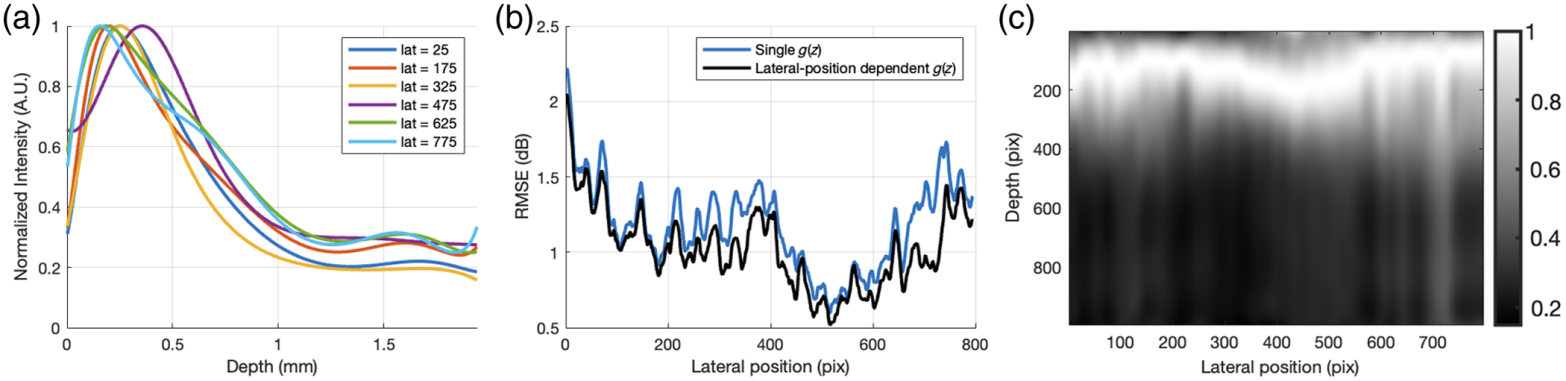
Comparison of basis and moving Basis Fit method. Panel (a) is an overlay of six extracted g^(z) at lateral positions 25, 175, 325, 475, 625, and 775 pixels. Panel (b) is the L2-norm RMSE between uncorrected images and corrected images by Basis Fit with a single g^(z) and with laterally-dependent g^(z). Panel (c) is g^(z) across all lateral positions, displayed as an image. White corresponds to the peaks of g^(z) with the greatest intensity. The pixel per mm conversion in the depth and lateral axis are 0.0020 and 0.0059 mm, respectively, assuming a refractive index of 1.

To investigate the extent to which g(z) is lateral-position-dependent in the B-scan, the A-scans within a moving window 25 pixels wide were used to produce a series of combined functions, assigned to the center of the window. Figure 10(a) shows an overlay of six extracted combined functions for windows centered at lateral positions 25, 175, 325, 475, 625, and 775 pixels. The combined functions at each lateral position were used to correct their corresponding A-scan. Each B-scan consisted of 795 lateral A-scans. The window size was 25 pixels. To correct the A-scans to the left of the center point of the first window, we used g^(z) extracted from the first window (pixels 1 to 25) to correct the A-scans at lateral positions 1 to 12. Similarly, g^(z) obtained from the last window (pixels 771 to 795) was used to correct the A-scans at lateral positions 784 to 795. Figure 10(b) shows a slight improvement in the RMSE of the corrected A-scans across all lateral positions, where the average for the black curve of 1.03 was lower than that of the blue curve of 1.18. Note that these values are normalized with respect to the maximum value of the blue curve in Fig. 10(b). Figure 10(c) shows the g^(z) for all A-scans at different lateral positions displayed as a grayscale image. The peaks of g^(z), shown by the white regions, is found to vary across the sample and correlates with the shape of the surface from the original B-scans, supporting the idea that the sample affects the shape and position of the confocal function.

**Fig. 11:**
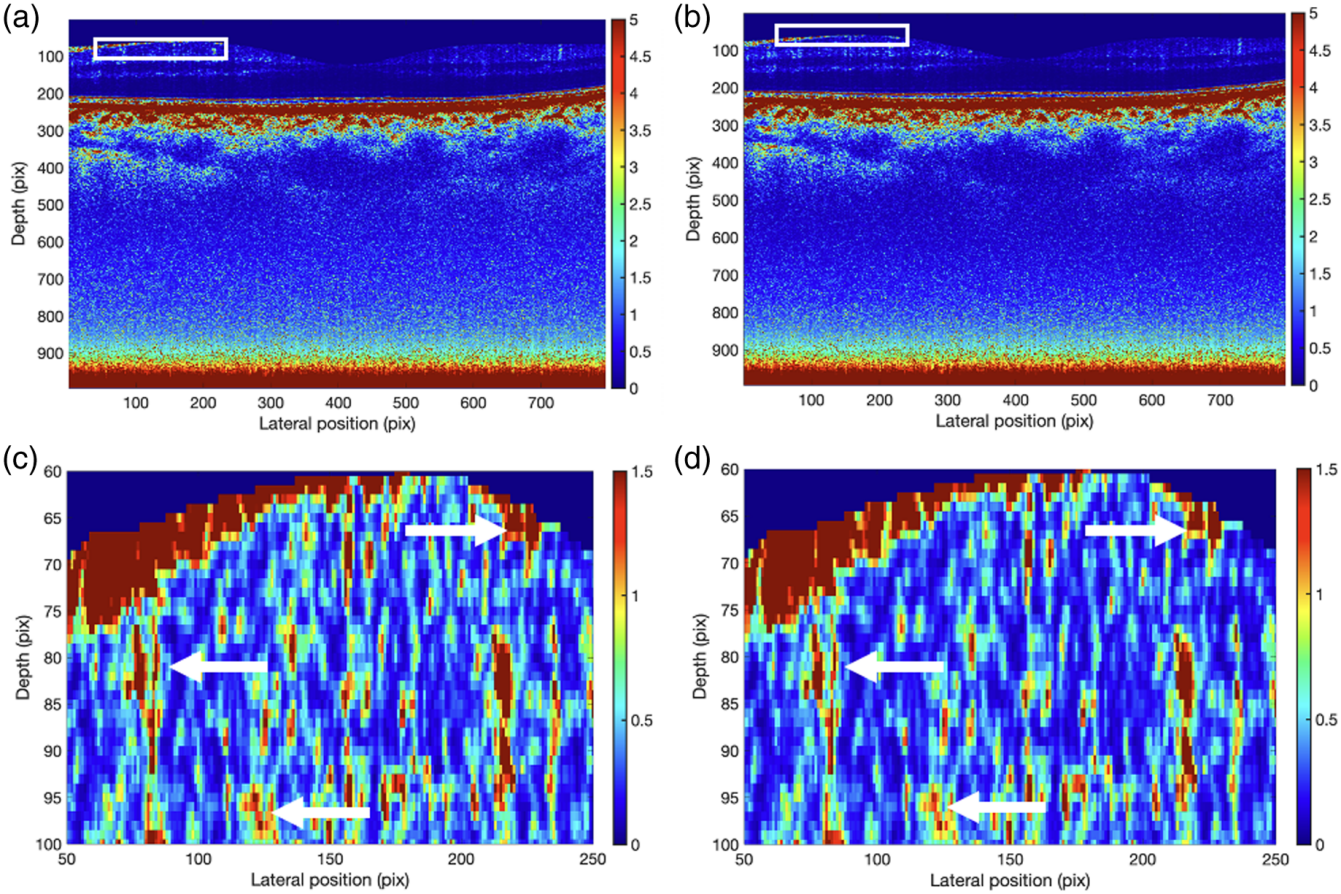
AC images of human retina. Panels (a) and (b) are from the corrected B-scans by Basis Fit and Ratio Fit, respectively. The pixel per mm conversion in the depth and lateral axis are 0.0020 and 0.0059 mm, respectively, assuming a refractive index of 1. Panels (c) and (d) are zoomed-in AC images of the white boxed regions. The white arrows highlight examples of differences between the two images, where (c) more pronounced spots of red than panel (d), indicating higher values obtained with Basis Fit.

From the corrected B-scans, the AC images were obtained by the DRC method. The resultant AC images after correction by the two different g^(z) extracted by Basis Fit and Ratio Fit are shown in Figs. 11(a) and 11(b), respectively. The differences between them can be seen more clearly in the zoomed-in images in Figs. 11(c) and 11(d) at the boxed regions. Compared with Ratio Fit of Fig. 11(c), the mean of the AC values of the zoomed-in image obtained by Basis Fit of Fig. 11(d) increased by 13%, indicating higher AC values directly below the ganglion cell layer of the retina, suggesting that Ratio Fit may underestimate the true AC in this region. In fact, it is known that the shape of the confocal function is sample-dependent^3^; therefore, this error in Ratio Fit is likely present in all biological samples.

## 6 Conclusion

As shown in simulation and images of phantom and biological samples, Basis Fit is able to extract the confocal and fall-off function as a single combined function, instead of extracting them separately, from two vertically shifted A-scans at the same lateral position (or B-scans). This method is also the first time that a linear combination of basis functions is used to model the combined function.

Basis functions eliminate the need to assume a specific form of the confocal function. Compared with other existing methods, the model-to-data fit can be significantly improved, especially when there is a mismatch between the actual and the assumed standard form of the confocal function that these methods employ. Given a sufficient number of basis functions, Basis Fit extracts from two B-scans of a vertically shifted sample a combined confocal and fall-off function that produces the smallest possible difference between corrected B-scans when the images are aligned, which is the goal of the original Ratio Fit method. In addition, Basis Fit robustly corrects images even when only a small shift is present between them.

A caution when using this method is if too many basis functions are used, the matrix B defined in Eq. (22) can become ill-conditioned, leading to poor extraction of the combined function. However, the situation can be remedied by reducing the number of basis functions or by using more B-scans of different sample depths. This method also makes several assumptions. Currently, the method assumes that there is no aliasing in the imaging caused by light reflecting back from beyond the ranging depth, and thus, it requires that the signal be fully attenuated within the ranging depth. In addition, this method assumes that the two B-scans used to extract the confocal and fall-off functions share the same confocal and fall-off functions and the only change is that the sample is vertically shifted. Thus, the method is applicable when the sample or OCT system can be vertically moved relatively close to each other, with no other translational shifts or rotations in other directions. As previously shown in Ref. 6, it is possible to extend this current method to accommodate horizontal translation and rotation in addition to vertical translation. The combined confocal and fall-off function found by this method can be applied to correct the interferogram or the windowed A-scans in spectroscopic OCT applications.

Finally, it is expected that the improvement in the extraction of the combined confocal and fall-off effects by this method should lead to improved medical diagnosis through more accurate AC images. In addition, this method enables future applications of OCT where precise removal of all depth-dependent effects (beyond the known confocal and fall-off effects) on OCT images is critical.

## Data Availability

Available upon reasonable request to the corresponding author.
